# Isolated locoregional recurrence patterns of breast cancer after mastectomy and adjuvant systemic therapies in the contemporary era

**DOI:** 10.18632/oncotarget.5365

**Published:** 2015-09-23

**Authors:** Jinli Ma, Rui Jiang, Lihua Fan, Xin Mei, Zhaozhi Yang, Xiaoli Yu, Xiaomao Guo, Zhen Zhang, Zhimin Shao

**Affiliations:** ^1^ Department of Radiation Oncology, Fudan University Shanghai Cancer Center, Shanghai, China; ^2^ Department of Radiation Oncology, Jingjiang People's Hospital, Jingjiang, China; ^3^ Department of Breast Surgery, Fudan University Shanghai Cancer Center, Shanghai, China; ^4^ Department of Oncology, Shanghai Medical College, Fudan University, Shanghai, China

**Keywords:** breast cancer, mastectomy, isolated locoregional recurrence, recurrence pattern, biologic subtype

## Abstract

**Purpose:**

To evaluate the recurrence patterns in a series of patients who presented with isolated locoregional recurrences (ILRRs) after mastectomy and adjuvant systemic therapies in the contemporary era.

**Methods:**

A total of 235 patients who developed ILRRs between 2005 and 2013 were classified into subgroups based on nodal status, hormone receptor status, and biologic subtype. The annual frequency of recurrences, association between biologic subtype and interval to recurrence (ITR), and anatomical distribution were evaluated.

**Results:**

For the entire group, recurrence peaked within the first 3 years after mastectomy, and then decreased significantly with time. Node-positive patients were observed to recur early, and a greater proportion recurred within 5 years (86.7% vs. 72.8%, χ^2^ = 6.83, *P* = 0.008) than did node-negative subgroup. Overall, the median ITR was 33.2 (range, 4.5 – 236) months. Biologic subtype specific median ITR were 43.3 (7.9 – 236.0) months for luminal A, 42.2 (6.1 – 143.3) months for luminal B, 23.8 (6.9 – 47.3) months for luminal HER2, 18.2 (6.6 – 117.5) months for HER2, and 21.8 (4.5 – 138.2) months for TNBC, and their difference was statistically significant (χ^2^ = 7.4, *P* = 0.001). Among all ILRRs, 51.5% (*n* = 121) were isolated to regional nodes.

**Conclusions:**

We demonstrates that the time course is consistent with previous description, biologic subtype is associated with ITR, and regional nodes is the most common place for recurrences in this series of patients who developed ILRRs following mastectomy and contemporary adjuvant systemic therapies but without PMRT.

## INTRODUCTION

Following mastectomy for operable breast cancer, approximately 5–10 percent of patients will develop a chest wall or regional recurrence within 10 years, and are subsequently at an increased risk for distant metastasis and death [1, 2]. Although a number of previous studies have reported the time course and anatomical distribution of locoregional recurrences (LRR) [3–8], the recurrence patterns might have changed with the advances in diagnostic technologies and therapeutic approaches in the adjuvant setting, and might vary with the population (e.g., patient subgroups) and time period studied. Recent studies have stratified breast cancers into five subtype-approximations defined by receptor expression and tumor grade [9–11]. These biologic subtypes are increasingly recognized as predictors of disease control following initial treatment; however, the implications of subtype in the recurrence patterns are less well studied.

The purpose of this study is to determine the frequency of recurrences over time, to examine the association between interval to recurrence (ITR) and constructed biologic subtype, and to evaluate the anatomical distribution of recurrences in a series of breast cancer patients who developed isolated LRR (ILRR) as a first event after initial treatment with mastectomy and adjuvant chemotherapy +/− endocrine therapy but without post-mastectomy radiation therapy (PMRT) in the contemporary era.

## RESULTS

During the period of Jan 2005 to Nov 2013, a consecutive series of 235 patients in total were identified to be eligible for analysis. Among these, 85% (*n* = 200) were referred to our hospital to seek further treatments for their recurrences with their primary tumors centrally reviewed; and the rest (15%) had been operated in our hospital for their primary breast cancers, and had detailed primary pathology data.

All LRRs were detected by imaging studies and/or physical examinations. Of these, 92.3% (*n* = 217) were pathologically confirmed from surgical specimens (*n* = 77) or fine needle aspirations (FNA) (*n* = 140); and the others were hard to be biopsied and diagnosed clinically based on radiological findings, consisting of 15 PET-CT scans and 3 contrast-enhanced CT scans.

### Patient characteristics

Patient characteristics of the entire group at the time of initial diagnosis are listed in Table 1. The median age was 47 years (range, 27 – 83 years) old. Of these, 59.1% (*n* = 139) were pre- and 40.9% (*n* = 96) post-menopausal, 55.3% (*n* = 130) had left- and 44.7% (*n* = 105) right-sided lesions, 86.8% (*n* = 204) underwent modified radical mastectomy (MRM), and the remaining (*n* = 31) underwent radical mastectomy (RM). Most patients had primary tumor (T) classification of pT1–2 (*n* = 215; 91.5%), and most tumors were moderately-poorly differentiated (*n* = 218; 92.8%). The median number of nodes examined was 12 (range, 6 – 30), and the distribution of the nodal stages was pN0, pN1, and pN2–3 in 54.9% (*n* = 129), 33.6% (*n* = 79), and 11.5% (*n* = 27) of patients, respectively. Constructed biologic subtypes consisted of luminal A in 35.3% (*n* = 83), luminal B in 24.7% (*n* = 58), luminal HER2 9.8% (*n* = 23), HER2 in 9.4% (*n* = 22), and TNBC in 20.9% (*n* = 49) of patients, respectively.

**Table 1:** The clinico-pathologic characteristics of patients at time of initial diagnosis (*n* = 235)

Parameters		Value
Median age (range) (years)		47 (27–83)
Interval to recurrence from mastectomy (mos)		33.2 (4.5–236)
Menopausal status	Pre- Post-	139 (59.1%) 96 (40.9%)
Location of primary tumor	Medial Central Outer	88 (37.4%) 12 (5.1%) 135 (57.3%)
Primary tumor histopathology	IDC Others	222 (94.5%) 13 (5.5%)
Primary tumor stage	pT1 pT2 pT3 Unknown	76 (32.3%) 139 (59.1%) 15 (6.4%) 5 (2.1%)
Tumor grade	1 2 3	17 (7.2%) 114 (48.5%) 104 (44.3%)
No. of nodes removed		12 (6–30)
Nodal stages	pN0 pN1 pN2 pN3	129 (54.9%) 79 (33.6%) 19 (8.1%) 8 (3.4%)
ER status	Positive Negative	149 (63.4%) 86 (36.6%)
PR status	Positive Negative	130 (55.3%) 105 (44.7%)
HER2 status	0~+ ++ +++	167 (71.1%) 32 (13.6%) 36 (15.3%)
Biologic subtype	Luminal A Luminal B Luminal HER2 HER2 Triple negative	83 (35.3%) 58 (24.7%) 23 (9.8%) 22 (9.4%) 49 (20.9%)
Primary surgery	MRM Others	204 (86.8%) 31 (13.2%)
Initial systemic therapy	Chemotherapy only Endocrine therapy only Chemotherapy + endocrine therapy Trastuzumab None	88 (37.4%) 11 (4.7%) 130 (55.3%) 3 (1.3%) 6 (2.6%)

Abbreviations: ILRR = isolated locoregional recurrence; IDC = invasive ductal carcinoma; ER = estrogen receptor; PR = progesterone receptor; HER2 = human epidermal growth factor receptor 2; MRM = modified radical mastectomy

The vast majority of patients had received some kind of adjuvant systemic therapy for their initial diagnosis of breast cancer, consisting of chemotherapy alone in 37.4% (*n* = 88), endocrine therapy alone in 4.7% (*n* = 11), and a combination of the two in 55.3% (*n* = 130) of patients. The median number of cycles of adjuvant chemotherapy was 6 (0 – 12), and most regimens were anthracycline- and/or taxane-based (*n* = 183). The endocrine therapies consisted of tamoxifen (TAM) alone in 113 patients, aromatase inhibitor (AI) alone in 20 patients, and TAM followed by AI in 8 patients. And very few patients (*n* = 3) were given anti-HER2 therapy.

### Frequency of ILRRs over time

**Figure 1:**
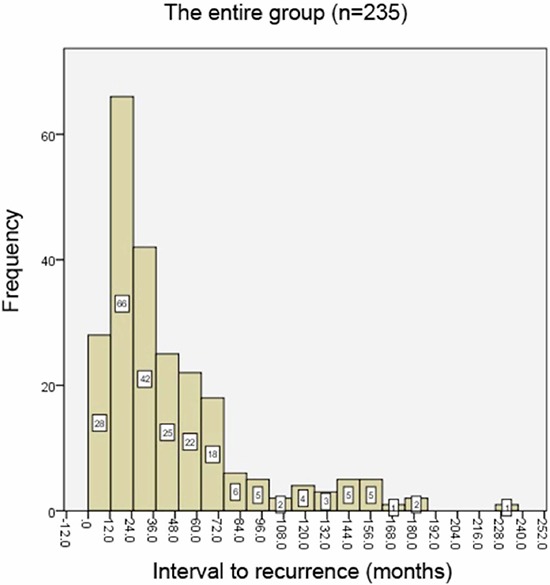
The annual frequency of isolated locoregional recurrences (ILRRs) for the entire group

**Figure 2:**
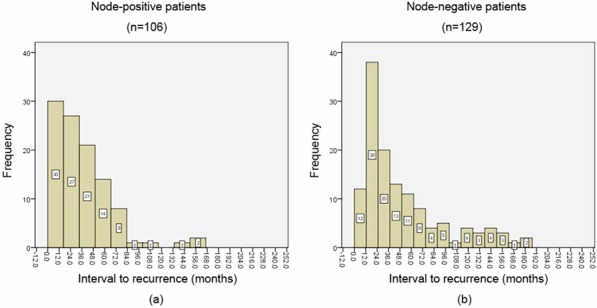
The annual frequency of isolated locoregional recurrences (ILRRs) for patient subgroups divided by nodal status

**Figure 3:**
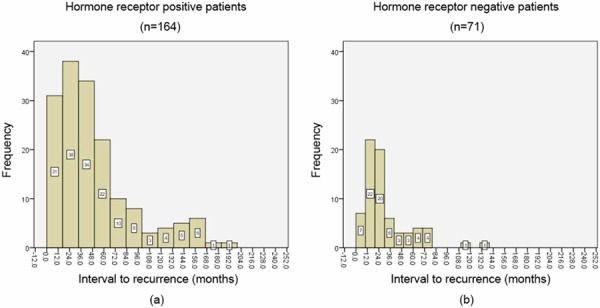
The annual frequency of isolated locoregional recurrences (ILRRs) for patient subgroups divided by hormone receptor (HR) status

Figure 1 showed the annual frequency of ILRRs for the entire group. The recurrence peaked within the first 3 years after surgery, and then decreased significantly with time. Around 80% of recurrences arose within 5 years. Figures 2 and 3 showed the annual frequency of recurrences for patient subgroups divided according to nodal status and hormone receptor (HR) status, respectively. Basically, the peak of disease recurrence in the initial 3 years persisted in all subgroups; however, there existed some major differences in terms of frequency of recurrences between subgroups. In comparison with node-negative patients, node-positive patients were observed to recur early following mastectomy, and a greater proportion recurred within 5 years (72.8% vs. 86.7%, χ^2^ = 6.83, *P* = 0.008). Compared with HR-positive subgroup, a smaller proportion of patients in the HR-negative group were observed to experience recurrences after 5 years; however, their difference did not reach statistical significance (23.2% vs. 14.1%, χ^2^ = 2.51, *P* = 0.112).

### ITR and its association with patient subgroup

For the entire group, the median ITR was 33.2 (range, 4.5 – 236) months. For node-negative patients (*n* = 129), the median ITR was 35.2 (4.5 – 186.0) months, compared to 30.7 (4.9 – 236.0) months for node-positive (*n* = 106) patients (χ^2^ = 10.5, *P* = 0.02). The subgroup of HR-positive patients (*n* = 164) had a median ITR of 36.9 (6.1 – 236.0) months, which is longer than in the subgroup of HR-negative (*n* = 71) patients (21.4 (4.5 – 138.2) months) (χ^2^ = 14.6, *P* < 0.001). Among node-negative patients (*n* = 129), the subgroup of HR-positive (*n* = 90) patients still had a longer median ITR than did HR-negative (*n* = 39) patients (39.1 (9.3 – 186.5 vs. 21.4 (4.5 – 138.2) months) (χ^2^ = 10.2, *P* = 0.003).

Overall, biologic subtype specific median ITR were 43.3 (7.9 – 236.0) months for luminal A (*n* = 83), 42.2 (6.1 – 143.3) months for luminal B (*n* = 58), 23.8 (6.9 – 47.3) months for luminal HER2 (*n* = 23), 18.2 (6.6 – 117.5) months for HER2 (*n* = 22), and 21.8 (4.5 – 138.2) months for TNBC (*n* = 49), and the difference among them was statistically significant (χ^2^ = 7.4, *P* = 0.001). Further, among node-negative patients (*n* = 129), biologic subtype specific median ITR were 56.8 (9.3 – 186.5) months for luminal A (*n* = 38), 36.1 (10.5 – 143.3) months for luminal B (*n* = 38), 31.8 (10.9 – 47.3) months for luminal HER2 (*n* = 14), 15.2 (8.1 – 117.5) months for HER2 (*n* = 11), and 21.6 (4.5 – 138.2) months for TNBC (*n* = 28), and their difference was statistically significant as well (χ^2^ = 7.1, *P* = 0.001).

### Anatomical distribution of ILRRs

**Figure 4:**
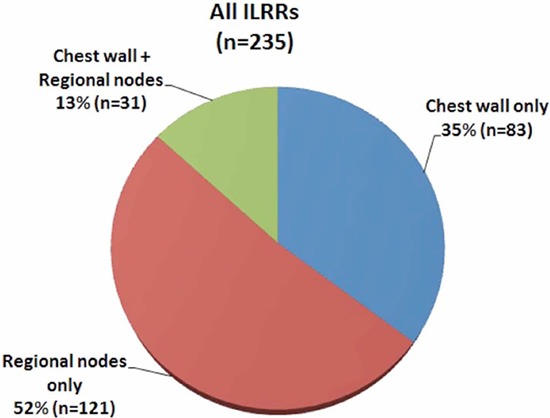
Overall distribution of isolated locoregional recurrences (ILRRs)

**Figure 5:**
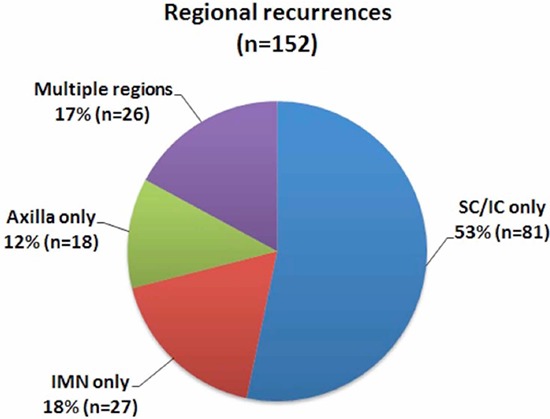
Distribution of regional recurrences

Among all ILRRs, 35.3% (*n* = 83) were isolated to the chest wall, 51.5% (*n* = 121) were isolated to regional nodes, and 13.2% (*n* = 31) occurred in both chest wall and regional nodes (Figure 4). Thus, 48.5% (*n* = 114) of patients had local recurrences, whereas 64.7% (*n* = 152) of patients had regional recurrences. Of regional recurrences, 53.3% (*n* = 81) were isolated to the supra-/infra-clavicular region, 17.8% (*n* = 27) were isolated to the internal mammary nodes (IMNs), 11.8% (*n* = 18) were isolated to the level I-II axilla, and 17.1% (*n* = 26) occurred in multiple regions (Figure 5). Overall, there were 44.7% (*n* = 105), 18.3% (*n* = 43), and 14.0% (*n* = 33) of patients relapsed in the para-clavicular region, IMNs, and level I-II axilla, respectively.

## DISCUSSION

The patterns of disease recurrence according to breast cancer characteristics and subtypes are of upmost importance to adequately inform patients and physicians about prognosis and also to guide planning of clinical trials. The description by Saphner et al. [8] of different patterns of recurrence for patients diagnosed from 1978 to 1988 has been a mainstay of clinical practice and research, particularly the discreet outcomes between estrogen-sensitive and estrogen insensitive tumors. However, since 1990s both diagnostic and therapeutic approaches to breast cancer have changed radically, and accordingly the overall outcomes of breast cancer patients have been improved [12]. Recently, breast cancer has been classified according to molecular factors that predict response to treatment. Cossetti et al. [13] demonstrated that the timing and patterns of recurrence differ according to breast cancer molecular subtypes and that these patterns persist with current therapies, albeit with better overall outcomes.

This analysis evaluated the recurrence patterns, e.g., time course, association between ITR and patient subgroup, and anatomical distribution, in a consecutive series of patients who presented with ILRRs after treatment with mastectomy and adjuvant systemic therapies in the contemporary era.

### Time course

In this analysis, annual frequency of recurrences, rather than annual hazard of recurrence (HRR), was used to determine the time course, due to the fact that this is not a cohort study dealing with recurrence risk in patients with diagnosis of breast cancer. The peak of recurrence for the entire group was demonstrated to be within the second year after surgery, which is consistent with the results of Saphner et al. [8] and others [13]. The pattern of a recurrence peak during the first 3 years was also noted to varying degrees in most subgroups based on nodal and HR status. However, node-positive patients showed an earlier peak of recurrence, i.e., approximately within the first year after mastectomy, and much less recurred beyond 5 years than did node-negative patients. This observation is consistent with those of Saphner et al. [8], who observed that the patients with the greater tumor burdens tend to have the steeper decreases in risk of recurrence. Similarly, when compared with HR-negative patients, a HR-positive status was associated with a greater proportion of recurrences occurring 5 years after surgery. In other words, HR-positive tumors recurred more frequently in late follow-up. This information may suggest that HR status embodies an entirely different natural history of breast cancer.

### The association of ITR and patient subgroup

We demonstrated a median ITR of 33 months for the entire group, which is roughly similar to previous reports. The analyses by nodal and HR status show that node-negative and HR-positive patients take a longer interval to develop recurrences. This observation provides further evidence supporting the above-mentioned findings that node-negative and HR-positive subgroups experienced more frequency of recurrences in late follow-up.

Further, our analysis demonstrated that an association existed between constructed biologic subtype and median ITR following mastectomy. The HER2 and TNBC patients, namely HR-negative subtypes known to be more aggressive, are shown to have shorter ITRs. This phenomenon might be attributed to the fact that the administration of adjuvant endocrine therapy delays disease recurrence in patients with node-negative, HR-positive breast cancer. Among all biologic subtypes, HER2 rather than TNBC patients are demonstrated to have the shortest ITRs. The reason for this outcome might be that in our study some of the HER2 patients were treated before the standard anti-HER2 treatment, and some could not afford the expenses and had to give up the use of adjuvant trastuzumab. Undoubtedly, the prognosis of HER2-positive patients has been improved with the use of trastuzumab [14–16]. The biology inferred by breast cancer subtypes therefore seems to be the major driver of disease recurrence. Whether the post-mastectomy follow-up frequency should be adjusted or not according to the specific recurrence pattern of different biologic subtypes is worthy to be discussed. For node-positive and HR-negative patients, a more frequent follow-up plan, especially during the first 5 years following mastectomy, might be warranted to facilitate early detection of recurrence.

### Anatomical distribution of ILRRs

Our analysis showed that a greater proportion (≈1/2) of recurrences were isolated to regional nodes. Chest wall and para-clavicular region were demonstrated to be involved in a similar proportion of the entire group. In addition, IM involvement was seen in 18% of patients. However, in review of previous series that report LRRs, around 60% are seen in chest wall only and 30% regional only (Table 2) [3–6]. When multiple recurrences are included, the chest wall may be involved in as many as 60–80% of patients, and the para-clavicular region involved in around 20–30% of patients in previous series (Table 3) [4–6, 17]. Besides, the involvement of IMNs in previous studies is not as common as our findings for this group of patients. Possible reasons for these differences in anatomical distribution of recurrences between this and previous series might include the thickness of flap left at surgery, era of adjuvant systemic therapies, and use of radiological imaging, etc. However, such an observation doesn't seem to change current practice on PMRT. In other words, ipsilateral chest wall and para-clavicular region remain the principle clinical target volume (CTV) to be irradiated in high risk breast cancer patients treated with mastectomy. Since inconsistent results were reported regarding the overall survival benefit from IM irradiation following mastectomy [18, 19], now is not the time to terminate the debate on IMN irradiation, though some major randomized trials evaluating the role of regional nodal irradiation did have included IMN as a part of CTV [20, 21]. Obviously, a case-by-case consideration of the risk of IMN nodal involvement, as well as patient anatomy and ability to exclude critical normal structures from the treatment fields, is imperative.

**Table 2:** Distribution of ILRRs after mastectomy by site

Study	No. of patients	Chest wall only	Regional nodes only	Chest wall + regional nodes
Nielsen et al. DBCG 82b/82c 2006	456	209 (46%)	197 (43%)	50 (11%)
Kuo et al. Taiwan 2008	115	69 (60%)	40 (35%)	6 (5%)
Skinner et al. M.D. Anderson 2013	159	100 (63%)	36 (23%)	23 (14%)
Shenouda et al. MGH 2014	103	67 (65%)	33 (32%)	3 (3%)
Current study	235	83 (35%)	121 (52%)	31 (13%)

Note: Total = 100%

**Table 3:** Distribution of ILRRs after mastectomy by site^#^

Study	No. of pts	Chest wall	SC/IC	IM	Axilla
Schwaibold, et al. UPenn 1991	128	106 (83%)	30 (25%)	4 (3%)	14 (7%)
Nielsen et al. DBCG 82b/82c 2006	456	259 (57%)	63 (14%)	NS	205 (45%)
Kuo et al. Taiwan 2008	115	75 (65%)	29 (25%)	NS	17 (15%)
Skinner et al. M.D. Anderson 2013	159	123 (77%)	43 (27%)	7 (4%)	20 (13%)
Current study	235	114 (49%)	105 (45%)	43 (18%)	33 (14%)

Note:

# indicates that multiple recurrences are included; Total > 100% because of inclusion of patients with multiple recurrence site

Abbreviations: SC/IC = supra/infra-clavicular region; IM = internal mammary nodes

In summary, our study demonstrates that the time course of recurrences is basically consistent with previous description, constructed biologic subtype is associated with interval to recurrence, and regional nodes is the most common place for recurrences in this series of patients who developed ILRRs following treatment with mastectomy and contemporary adjuvant systemic therapies but without PMRT. Prospective studies to further investigate the implications of constructed biologic subtype and its relationship to recurrence patterns following mastectomy in these women are warranted.

## MATERIALS AND METHODS

After obtaining Fudan University Shanghai Cancer Center Institutional Review Board (IRB) approval for a retrospective chart analysis, the electronic medical records from an institutional database were retrospectively reviewed to screen for female breast cancer patients who presented with post-mastectomy ILRR and received definitive locoregional RT as an integral part of multimodal treatment. The ILRR is defined as a recurrence within the ipsilateral chest wall and/or regional nodes (i.e., ipsilateral axilla, supra-/infra-clavicular region, or internal mammary chain), without concomitant visceral or bony distant metastasis (DM) within 4 months of LRR. All metastases were ruled out by thorough restaging evaluation, e.g., brain MR, chest CT, abdomen US or MR, and ECT, etc. Patients who received previous radiation to the chest wall and/or regional nodes, or had pathology other than breast cancer, or with unknown receptor status, were excluded from this study.

Data were extracted from prospectively collected institutional database to determine clinical, pathologic, treatment, and outcome variables. Health information management professionals trained in data abstraction reviewed the complete medical records of eligible patients. Information, including age at diagnosis, menopausal status, stage, grade, histology, and biomarkers (i.e., estrogen receptor (ER), progesterone receptor (PR), and human epidermal growth factor receptor 2 (HER2)) were extracted. Data on systemic therapy (i.e., chemotherapy, anti-HER2 therapy, and hormonal therapy) and dates of diagnosis and disease recurrence were also obtained.

Patients were classified into subgroups based on the characteristics at time of initial diagnosis, e.g., nodal and HR status. Breast cancer biologic subtypes were constructed according to ER, PR, HER2 status, and tumor grade into 5 categories as follows: i) luminal A: ER+ or PR+, HER2-, and grade 1 or 2, ii) luminal B: ER+ or PR+, HER2-, and grade 3, iii) luminal HER2: ER+ or PR+, and HER2+, iv) HER2: ER- and PR- and HER2+, and v) triple negative breast cancer (TNBC): ER- and PR-, and HER2- [9, 10]. Basically, luminal A, B and luminal HER2 subtypes constitute HR-positive subgroup, whereas HER2 and TNBC subtypes constitute HR-negative subgroup.

ER and PR status were evaluated by immunohistochemistry (IHC) staining, and were considered positive if IHC staining ≥ 10% of tumor tissue. HER2 status was determined by IHC staining. Tumors were considered HER2 positive if they scored 3+, on IHC, indeterminate if 2+, and negative if 1+ or 0. When IHC was indeterminate, tumors were considered HER2 positive with amplification (ratio ≥ 2.0) by fluorescence *in situ* hybridization (FISH) analysis.

The ITR is defined as the length of time from the date of mastectomy until local and/or regional recurrence occurred. The annual frequency and anatomical distribution of recurrences was determined using descriptive statistics. The χ^2^ and Fisher's exact tests were used to compare the frequencies between patient subgroups. K Independent-Samples Test was used to evaluate the association between ITR and patient subgroups, e.g., constructed biologic subtype. All statistical tests were two-sided, and *P* < 0.05 was considered statistically significant. SPSS for Windows version 17.0 (SPSS, Chicago, IL) was used for all statistical analyses.
